# Compositional Variations in Wheat Bran Influence Growth Performance, Nutrient Retention, and Cecal Microbiome in Broilers

**DOI:** 10.3390/ani14233407

**Published:** 2024-11-26

**Authors:** Yujing Feng, Songjun Jiao, Ying Zhang, Youyou Liu, Feng Zhao, Yuming Wang, Renna Sa, Jingjing Xie

**Affiliations:** The State Key Laboratory of Animal Nutrition and Feeding, Institute of Animal Science, Chinese Academy of Agricultural Sciences, Beijing 100193, China; 16688208780@163.com (Y.F.); jsj2470978386@163.com (S.J.); zyzyyjs@163.com (Y.Z.); liu15jay@163.com (Y.L.); zhaofeng@caas.cn (F.Z.); wangyuming@caas.cn (Y.W.); sa6289@126.com (R.S.)

**Keywords:** wheat bran, chemical composition, total tract metabolizability of nutrients, cecal microbiota, broiler

## Abstract

Recent studies have discovered that wheat bran could stimulate digestive tract development, modulate intestinal microbiota, and improve the digestibility of fibrous ingredients in broilers. Wheat bran varies greatly in its chemical constituents. The objective of this study is to investigate how different types of wheat bran influence growth performance, nutrient digestion, and gut health in broiler chickens. Eight types of wheat bran were sampled and analyzed for nine constituents. A principal component analysis demonstrated that the wheat bran could be clustered into three groups based on the first principal component, namely low-fiber (LF), medium-fiber (MF), and high-fiber (HF) wheat bran. Wheat bran from the LF, MF, and HF groups significantly differed in terms of the contents of NDF, acid detergent fiber (ADF), hemicellulose, in vitro digestible energy (IVDE), crude fat, and ash (*p* < 0.05). A total of 324 seven-day-old broiler chicks were grouped into six body weight blocks. Each block of birds was randomly divided into six treatments, fed either a corn–soybean meal basal diet or five experimental diets, including 3% wheat bran from the LF, MF, and HF groups, until d 21. The feed/gain ratio was increased, and the coefficients of dry matter (DM, *p* = 0.001), gross energy (GE, *p* = 0.005) digestion, energy (*p* = 0.039), and CP (*p* = 0.051) retention were reduced in the birds that were fed 3% HF wheat bran. In contrast, in the birds fed 3% LF wheat bran, the coefficients of nutrient digestion and retention of energy and CP were not affected, but the CP (*p* = 0.009) and the NDF (*p* = 0.059) digestion ability were increased when switched to the same diet. In the cecal digesta, *Barnesiella* was enriched in the birds from the LF group, while *Lachnospiracea* was enriched in the birds from the MF group. Adding a moderate amount of wheat bran from the LF group could promote protein and fiber digestion and increase the abundance of *Barnesiella* in broilers.

## Simple Summary

Wheat bran is a cost-effective feed ingredient that can improve growth performance and intestinal infection resistance in broiler chickens when used in moderation. Due to different milling processes, wheat bran varies significantly in its chemical constituents. It remains unclear how these chemical variations in wheat bran relate to its nutritional functions. This study aims to identify criteria to categorize wheat bran based on its proximate components and in vitro digestible energy and further determine the differences in growth performance, nutrient digestion, and gut health among different groups of wheat bran. The results show that including low-fiber (LF) wheat bran in feed increased broilers’ crude protein (CP) capacity and fiber digestion and promoted the growth of *Barnesiella*, a genus regulating IFN-γ production, but did not affect growth performance. In contrast, high-fiber (HF) wheat bran in feed decreased the gross energy (GE) and CP retention. Therefore, wheat bran containing less than 40% neutral detergent fiber (NDF) is recommended for use in broiler diets, representing a promising way to decrease feed costs and improve gut health.

## 1. Introduction

Wheat bran is a by-product of milling grains into flour. It mainly consists of the pericarp, seed coat, and aleurone layers of wheat grains and is a rich source of insoluble fiber [1]. Insoluble fiber has long been considered an anti-nutritive component in poultry diets; however, adding a moderate amount of insoluble fibers such as oat and sunflower hull to broiler diets can promote the development of gizzard. It is known that insoluble fibers can improve the digestion of dietary nutrients, the feed intake, and the feed conversion ratio [2,3,4,5]. Adding 3% wheat bran to a broiler diet has been reported to promote gizzard development, amylase and trypsin activity, and the digestion of dietary nutrients in several studies [3,6].

Approximately 60% of the dietary fiber fraction in wheat bran is arabinoxylan. Xylo-oligosaccharides derived from arabinoxylan can selectively stimulate the growth of intestinal microorganisms, such as *Lactobacillus*, *Butyraticus*, and *Akkermansia* and increase the production of short-chain fatty acids (SCFAs) in broilers [7,8]. Owing to its prebiotic potential, a small amount of wheat bran has been previously included in poultry diets to fight against necrotic enteritis and *Salmonella* infection [9,10]. Recent studies have also shown that including wheat bran in starter feed can kick-start the digestion of sorghum [11] and barley [6,12] by modulating the intestinal microbiota. Therefore, the dietary inclusion of wheat bran is a promising means to improve broilers’ growth performance by improving the utilization of high-fiber feed ingredients and intestinal health.

Due to different processes of milling flour, the chemical constituents of wheat bran vary greatly, such as the crude protein (CP), crude fiber (CF), and ash content [1]. According to data from Feedpedia (https://feedipedia.org/, accessed on 16 November 2022), wheat bran consists of approximately 14.1–20.5% CP, 11.1–35.4% starch, 6.3–14.7% CF, 32.4–56.5% neutral detergent fiber (NDF), and 8.4–17.6% acid detergent fiber (ADF) [13]. Chemical composition determines the nutritional value of wheat bran. From the standpoint of effective energy, Ning et al. [14] showed that the net energy of the wheat bran fed to broiler chickens was negatively correlated with the crude fiber, CP, and ADF. So far, little information is available on how compositional variations in wheat brans influence the digestive function and gut health of broilers. Thus, the objective of the current study is to investigate the effects of wheat bran with different chemical compositions on growth performance, nutrient digestion, and intestinal microbiota to identify the chemical variables that can predict its beneficial functions.

## 2. Materials and Methods

### 2.1. Chemical Analyses of Wheat Bran

Eight wheat bran samples were obtained from the following: Beijing Hefeng Co., Ltd. (Beijing, China, WB1); Shandong New Hope Liuhe Group Co., Ltd. (Qingdao, China, WB2); Qingdao Haomianyuan Flour Co., Ltd. (Qingdao, China, WB3); Wutory Group Dongming Flour Co., Ltd. (Dongming, China, WB4); Yihai Yantai Grain and Oil Industry Co., Ltd. (Yantia, China, WB5); Shandong Linkun Flour Industry Co., Ltd. (Taian, China, WB6); Shandong Yuanhao Flour Co., Ltd. (Dongying, China, WB7); and the feed mill of the Institute of Animal Science, Chinese Academy of Agriculture Science (Beijing, China, WB8). All samples were analyzed for their dry matter (DM, method 930.15; [15]), starch (method 996.11; [15]), CP (method 968.06; [15]), ether extract (EE, method 920.39; [15]), NDF (method 2002.04; [15]), ADF [16], and ash (method 942.05; [15]). The hemicellulose content was calculated as the difference between the NDF and the ADF. The in vitro digestible energy (IVDE) was determined in a computer-controlled simulated digestion system, as described by Yu et al. [17], which has been demonstrated to accurately predict the apparent metabolizable energy (AME) in chickens. Principle component analysis (PCA) was conducted to identify the major contributors to the differences among wheat bran types, and the eight wheat bran samples were clustered into three groups, named the low-fiber (LF), medium-fiber (MF), and high-fiber wheat bran (HF), accordingly.

### 2.2. Animals, Diets, and Treatments

The procedure for the animal experiment was reviewed and approved by the Animal Welfare Ethics Committee in the Institute of Animal Science, Chinese Academy of Agriculture Science (IAS 2021-109). The feeding trial was conducted in a completely randomized block design. The corn–soybean meal basal diet was formulated to meet or exceed the nutritional requirements for Arbor Acres (AA) broilers [18]. The five additional experimental diets were obtained by replacing the corn and soybean meal in the basal diet with 3% wheat bran selected from the LF (WB1), MF (WB2, WB3), and HF (WB5, WB6) groups, respectively. All the experimental diets were formulated to be isocaloric (Table 1). The analyzed IVDE of all the ingredients were used to calculate the energy content of each diet.

Day-old AA male broiler chicks were purchased from a commercial hatchery (Beijing Huadu Broiler Co., Ltd., Beijing, China) and fed with the same starter feed from their arrival to d 6 in metabolic cages. All chickens were kept under the environmental conditions according to the breeder’s recommendations. The temperature inside the facility was set as 35 °C on d 0 and decreased by 1 °C every three days until the end of the experiment. A 24 h light schedule was used throughout the experimental period. Feces were removed every three days except for the excreta collection period. Feed and water were provided ad libitum. On d 7, a total of 324 birds were divided into 6 body weight (BW) blocks. Chicks from each block were then randomly assigned to one of the 6 treatment groups, with 9 birds in each group. Each group of chicks was either fed with the basal diet (CON) or one of the 5 experimental diets for 14 days. Nutrient digestibility was determined for each experimental diet. Digestion ability was determined by measuring the coefficients of nutrient metabolizability when all treatment groups switched to the same finisher diet from d 21 to d 28.

### 2.3. Sample Collection

Two rounds of total excreta collection were carried out on d 17 and d 24 for 72 h, respectively. The first excreta collection period started from 21:00 of d 17 to 21:00 of d 20 and the second collection period started from 21:00 of d 24 to 21:00 of d 27. The cumulative feed intake was recorded. All excreta were collected and stored at −20 °C. The excreta were dried in a conventional oven at 65 °C to a constant weight and ground through a 0.42 mm screen before chemical analyses.

On d 21, the BW was taken for each treatment group. One bird was randomly selected from each group and euthanized using CO_2_ to collect the cecal chyme. The chyme was snap-frozen in the liquid nitrogen and transferred to −80 °C for storage.

### 2.4. Chemical Analyses and Calculations

The contents of DM, gross energy (GE), CP, NDF, and ADF of diets and excreta were determined as mentioned above. The coefficients of total tract nutrient metabolizability were calculated using the following formula:

$$Coefficient\;of\;total\;tract\;nutrient\;metabolisability=1-\frac{nutrient\%\;in\;excreta\times Excreta\;output\;\left(g\right)}{nutrient\%\;in\;feed\times Feed\;intake\;\left(g\right)}$$

### 2.5. 16S rRNA Amplification, Sequencing, and Bioinformatic Analysis

The bacterial DNA was extracted from the cecal chyme samples using E.Z.N.A.^®^ soil DNA kit (Omega Bio-tek, Norcross, GA, USA). The extracted DNA was amplified in triplicates with the barcoded primer set (338F: 5′-ACTCCTACGGGAGGCAG-3′; 806R: 5′-GGACTACHVGGGTWTCTAAT-3′) covering the V3–V4 variable region of the bacterial 16S rDNA. The PCR product was recovered from 2% agarose gel and purified using the AxyPrep DNA Gel Extraction Kit (Axygen Biosciences, Union City, CA, USA), according to the manufacturer’s instructions. The DNA libraries were quantified using Quantus™ Fluorometer (Promega (Beijing) Biotech Co., Ltd., Beijing, China) and sequenced on Illumina’s Miseq PE300 platform. Raw sequences were quality controlled and filtered with the fastp software tool (version 0.19.6), and then spliced using the FLASh software tool (version 1.2.7). The DADA2 tool plug-in in the QIIME2 (version 2022.4) was used to denoise the sequences and bin to obtain the amplicon sequence variants (ASVs).

All bioinformatic analyses were performed on the analytic platform provided by Majorbio Bio-Pharm Technology Co., Ltd. (Shanghai, China). The Naive Bayes classifier in QIIME2 was used to annotate the ASVs against the Silva 16S rRNA gene database (version 138) and the chloroplast and mitochondrial ASVs were filtered. The α-diversity indices, such as Chao1, ACE, Shannon and Simpson, were calculated by the Mothur (version 1.30) software, and the differences in α-diversity indices among treatments were analyzed using the Kruskal–Wallis rank sum test. The principal coordinates analysis (PCoA) based on the Bray–Curtis dissimilarity metrics was conducted at the genus level and assessed using the ANalysis Of SIMilarity (ANOSIM). The compositional variations were tested at the phylum and genus level with the Kruskal–Wallis rank sum test and corrected with the false discovery rate (FDR), followed by the Welch’s test for the pairwise comparisons. The all-against-all strategy was used in the linear discriminant analysis effect size (LEfSe) to determine the taxonomic features for each treatment group with a linear discriminant analysis (LDA) score > 2 and *p* < 0.05. The Kyoto Encyclopedia of Genes and Genomes (KEGG) functions were annotated by the PICRUSt2 software (version 2.2.0-b) and Kruskal–Wallis rank sum tests were used to test the differences in carbohydrate enzymes (CAZymes) among different treatments. The datasets generated and/or analyzed during the current study were available in the National Center for Biotechnology Information (NCBI) repository, accession number PRJNA988507.

### 2.6. Analysis of SCFAs in the Cecal Digesta

Short chain fatty acids were extracted using distilled water. Approximately 1 g of cecal chyme sample was incubated with 5× volumes of the distilled water overnight at 4 °C. The supernatants were obtained by centrifuging at 2000× *g* for 10 min at 4 °C. The pellets were re-extracted with the same volume of the distilled water. The two supernatants were combined to make up to a known volume with the distilled water. A volume of 900 μL SCFA extract was thoroughly mixed with 100 μL of aqueous metaphosphoric acid (25%, *w*/*v*) and left to stand at the room temperature for 4 h. Then, the samples were centrifuged at 10,000 rpm for 10 min and the supernatants were passed through 0.22 μm filter before the gas chromatographic analyses. The Agilent 7890 gas chromatograph was equipped with a 30 m (0.25 mm) DB-FFAP column (Agilent, Wilmington, DE, USA). A 1:50 (*v*:*v*) split injector was used with the flame ionization detector, where the injection volume was set at 1 μL at 250 °C and the detection temperature was set at 280 °C. The carrier gas was nitrogen with a flow rate of 0.8 mL/min. The stocking solution of SCFA standards was prepared to contain 20.924 mg/mL acetate, 6.190 mg/mL propionate, 7.671 mg/mL butyrate, 1.058 mg/mL isobutyrate, 2.217 mg/mL isovalerate, and 1.920 mg/mL valerate in the distilled water. Serial dilutions of the stocking solutions were used to generate the calibration curve.

### 2.7. Statistical Analyses

The JMP Pro software (version 16.0, SAS Institute Inc., Cary, NC, USA) was used for statistical analyses, and the GraphPad Prism software (version 9.3.1, San Diego, CA, USA) was used for plotting figures. The row-wise Pearson correlations and the PCAs were performed on the multivariate statistical platform of JMP. The minimum Akaike information criterion method was used in the stepwise regression analyses to establish the equation with the best fit to estimate IVDE from chemical compositions. The data of growth performance and SCFAs were analyzed on the fitting model platform for Analysis of Variance (ANOVA) tests, with diet as the fixed effect and the BW block as the random effect. The contrast analyses were conducted to test the differences among different categories of wheat brans when the fixed effect of diet was significant. All data were presented as mean ± SEM. The statistical significance was considered when *p* < 0.05, and trends were considered to be significant when 0.05 ≤ *p* < 0.10.

## 3. Results

### 3.1. Chemical Compositions of Wheat Brans

**Figure 1:**
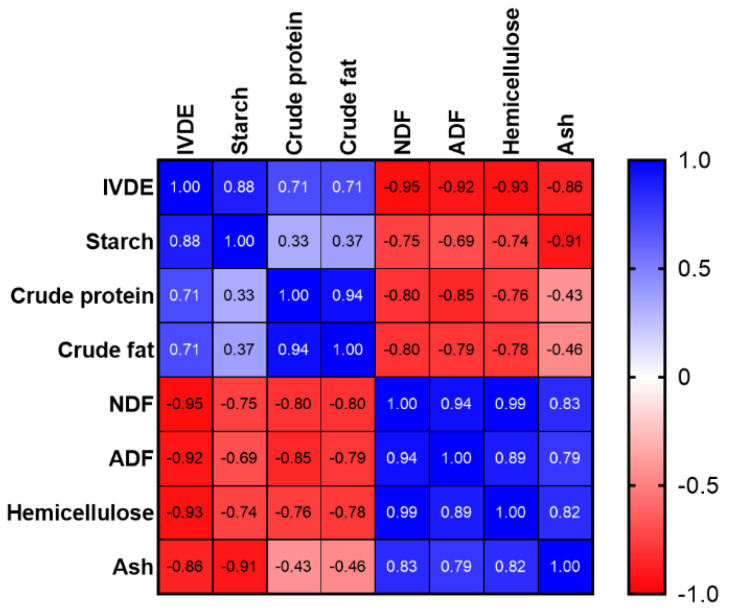
Pearson correlation analysis of in vitro digestible energy (IVDE) and proximate components of the wheat brans. Blue colors indicate positive correlations, while red colors indicate negative correlations. Different densities of color indicate *p* values. Abbreviations: IVDE, in vitro digestible energy; NDF, neutral detergent fiber; ADF, acid detergent fiber.

Among all the chemical constituents, the starch content had the greatest coefficient of variation (CV, Table 2). The CVs for the contents of NDF, ADF, and hemicellulose were around 19%. The IVDE of the eight samples of wheat bran ranged from 1751 to 2721 kcal/kg. The row-wise Pearson correlation analysis showed that the IVDE was positively correlated with the content of starch (*p* = 0.004), CP (*p* = 0.049), and EE (*p* = 0.050), but negatively correlated with the content of the NDF (*p* < 0.001), ADF (*p* = 0.001), hemicellulose (*p* = 0.001), and ash (*p* = 0.006, Figure 1). The equation with the best fit to estimate the IVDE from chemical variables of wheat bran was IVDE (kcal/kg DM) = 2966 + 23.65 × Starch − 26.60 × NDF.

**Figure 2:**
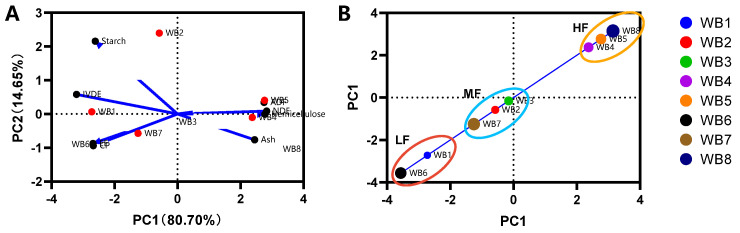
Principal component analysis (PCA) of chemical variables of wheat bran. (**A**) Biplot showing variation in the proximate components and in vitro digestible energy (IVDE) for the eight wheat brans. (**B**) Clusters of the eight wheat brans based upon PC1. Abbreviations: PC1, the 1st principal component; PC2, the 2nd principal component; IVDE, in vitro digestible energy; CP, crude protein; EE, ether extract; NDF, neutral detergent fiber; ADF, acid detergent fiber; LF, low-fiber content; MF, medium-fiber content; HF, high-fiber content.

The PCA of all nine chemical variables showed that the first and second principal components contributed 80.70% and 14.65% to the compositional variances of the eight wheat bran samples, respectively (Figure 2A). The first principal component (PC1) positively correlated with the content of the fibrous components (NDF, ADF, and hemicellulose), but negatively correlated with the content of the digestible nutrients (IVDE, starch, CP, and EE) (*p* < 0.05). According to the scores of the PC1, the eight wheat bran samples were clustered into three groups, named the LF, MF, and HF wheat bran groups, respectively.

**Figure 3:**
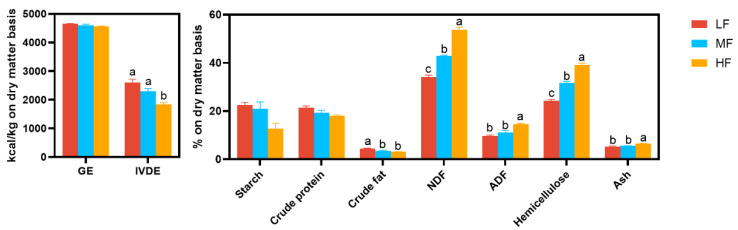
Compositional differences among wheat brans from different categories. Different letters indicate significant differences at *p* < 0.05. Abbreviations: GE, gross energy; IVDE, in vitro digestible energy; NDF, neutral detergent fiber; ADF, acid detergent fiber; LF, low-fiber content; MF, medium-fiber content; HF, high-fiber content.

As shown in Figure 3, there were significant inter-group differences in the IVDE (*p* = 0.004), and in the contents of NDF (*p* < 0.001), ADF (*p* = 0.005), hemicellulose (*p* < 0.001), crude fat (*p* = 0.031), and ash (*p* = 0.018). The contents of starch (*p* = 0.071) and CP (*p* = 0.076) in different groups of wheat bran tended to be different. The LF wheat bran had the lowest contents of NDF, ADF, hemicellulose, and ash but the highest IVDE and crude fat contents (*p* < 0.05). The contents of ADF, IVDE, and ash were comparable between the MF and the LF wheat bran (*p* > 0.05), but the NDF and hemicellulose contents were higher and the crude fat contents were lower in the MF wheat bran than in the LF wheat bran (*p* < 0.05).

### 3.2. Growth Performance

There were no significant differences in the BW on d 21 (*p* = 0.167) or in the average daily gain (ADG, *p* = 0.155) and the average daily feed intake (ADFI, *p* = 0.409) from d 7 to d 21 among all the treatment groups (Table 3). Except for the birds fed with WB1 wheat bran, the feed-to-gain ratio (F/G) was greater in the birds fed with 3% wheat bran than those fed with the basal diet (*p* = 0.007). Contrast analyses showed that the birds fed with the LF wheat bran had a lower F/G than those fed with the MF wheat bran (*p* = 0.039) and the HF (*p* = 0.069) wheat bran.

### 3.3. Total Tract Nutrients Metabolizability and Digestion Ability

On d 17–20, greater coefficients of the total tract metabolizability of DM (*p* = 0.001) and GE (*p* = 0.005) were noted in the birds fed with the basal diet than in those fed with WB3, WB4, and WB5 wheat bran (Table 4). But the coefficient of the CP metabolizability did not differ among all treatment groups. Over this period, higher retentions for GE and CP were found in the CON and the WB1 group than in the WB4 and WB5 groups, but the metabolizability coefficient of NDF was greater in the latter groups (*p* < 0.001). Contrast analyses revealed that the metabolizability coefficient of NDF in birds fed with the HF wheat bran was greater than in those fed with the LF (*p* = 0.001) wheat bran and the MF (*p* = 0.004) wheat bran.

The digestion ability was determined as the coefficients of total tract nutrients metabolizability when all treatment groups consumed the same diet. As shown in Table 5, the birds from the WB1 treatment had greater digestion ability for the CP (*p* = 0.009) and NDF (*p* = 0.050) than those on the CON, WB4, and WB5 treatments. Contrast analyses revealed that the metabolizability coefficients of the CP and NDF were higher in the LF group than in the HF (CP, *p* = 0.001; NDF, *p* = 0.008) and the MF group (CP, *p* = 0.073; NDF, *p* = 0.042). No differences were found in the digestion ability for the DM and ADF among the three wheat bran groups.

### 3.4. Cecal Microbiota

**Figure 4:**
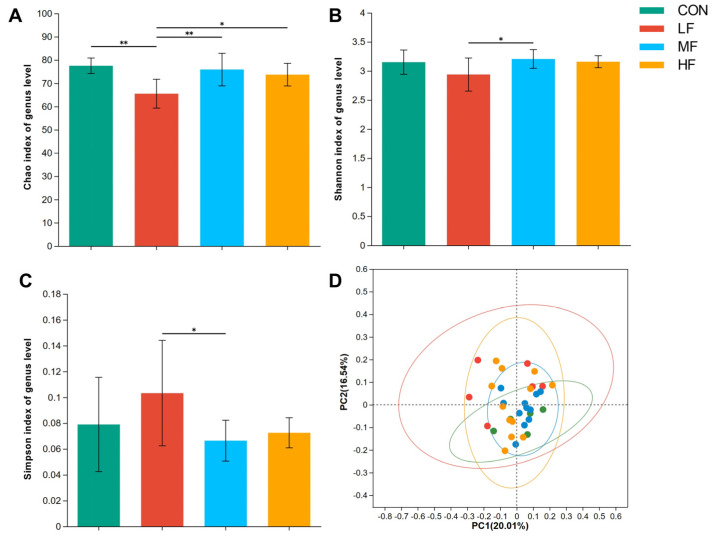
Alpha and beta diversity of cecal microbiota in 21-day-old broilers fed with different categories of wheat brans. (**A**) Chao1, (**B**) Shannon, and (**C**) Simpson indices of cecal bacterial community at the genus level. (**D**) PCoA analysis based on Bray–Curtis dissimilarity metrics. ** indicates significant differences at *p* < 0.05. * indicates significant differences at *p* < 0.01. Abbreviations: LF, low-fiber content; MF, medium-fiber content; HF, high-fiber content; PCoA, principal coordinate analysis; PC1, the 1st principal component; PC2, the 2nd principal component.

Owing to the inter-group differences in the digestion ability for NDF and CP among different treatments, the composition and function of cecal microbiota were determined. The data revealed that the Chao1 (Figure 4A), Shannon (Figure 4B), and Simpson (Figure 4C) indices of the cecal microbiota varied among all the treatment groups on d 21. As shown, the LF group had the lowest Chao1 and Shannon indices at the genus level, but the highest Simpson index (*p* < 0.05). The PCoA showed that the composition of the cecal microbiota was similar in the MF and HF groups, but differed between the CON and LF groups (*p* = 0.033, Figure 4D).

**Figure 5:**
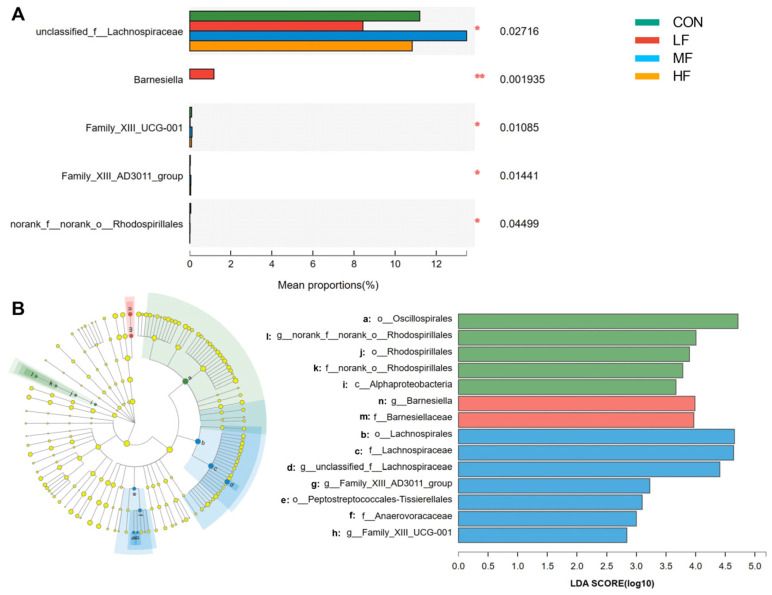
Cecal microbiota composition in 21-day-old broilers fed with different categories of wheat brans. (**A**) Differential abundant microbes among treatments. (**B**) Taxonomic features determined by LDA. * indicates significant differences at *p* < 0.05. ** indicates significant differences at *p* < 0.01. Abbreviations: LF, low-fiber content; MF, medium-fiber content; HF, high-fiber content; LEfSe, linear discriminant analysis effect size; LDA, linear discriminant analysis.

At the phylum level, Firmicutes, Bacteroides, and Proteobacteria comprised over 99% of the cecal microflora. At the genus level, *Faecalibacterium*, *Alistipes*, *Ruminococcus torques group*, *Bacteroides*, *Butyricicoccus*, *Lactobacillus*, and three unclassified genera from *Lachnospiraceae*, *Oscillospiraceae*, and *Clostridia UCG-014* were the top genera and comprised approximately 60% of the total microflora. Significant inter-group differences were noted in the unclassified *Lachnospiraceae*, *Barnesiella*, *Family XIII UCG-001*, *Family XIII AD3011 group*, and *Rhodospirillales* (Figure 5A). The LEfSe analyses (Figure 5B) revealed that *Oscillospirales* and *Rhodospirillales* from the α-proteobacteria family were enriched in the cecal microbiota of the CON group, *Barnesiella* were enriched in the LF group, and *Lachnospiracea*, *Family XIII UCG-001*, and the *Family XIII AD3011 group* from the Peptostreptococcales-*Tissierellale* were enriched in the MF group.

**Figure 6:**
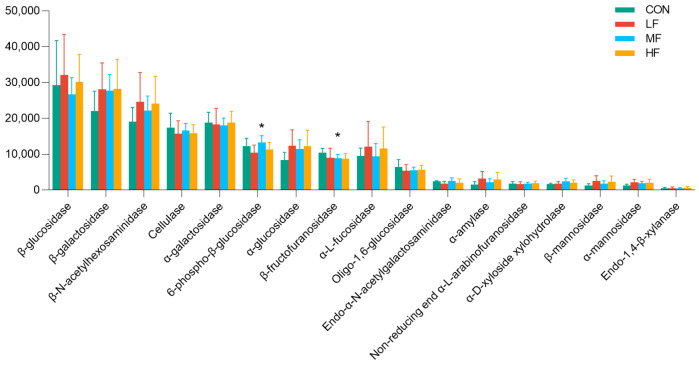
Abundance of glycoside hydrolase annotated by PICRUSt2. * indicates significant differences at *p* < 0.05. Abbreviations: LF, low-fiber content; MF, medium-fiber content; HF, high-fiber content.

Abundant glycoside hydrolase (GH), such as β-glucosidase, β-galactosidase, β-N-acetylhexosaminidase, cellulase, α-galactosidase, 6-phospho-β-glucosidase, α-glucosidase, β-fructofuranosidase, and α-L-fucosidase (Figure 6), were found in the genome of cecal microbiota community of 21-day-old broilers. Among these glycosidases, only the abundances of 6-phospho-β-glucosidase (*p* = 0.016) and β-fructofuranosidase (*p* = 0.040) were noted to be different among the treatment groups. Compared to the control birds, the expression of β-fructofuranosidase was significantly higher in the MF wheat bran group (*p* = 0.040), whereas the expression of 6-phospho-β-glucosidase was higher in the MF group than in the HF and LF groups (*p* = 0.016).

### 3.5. Cecal SCFAs

The contents of acetate, propionate, isobutyrate, butyrate, isovalerate, valerate, and total SCFA in the cecal digesta did not show significant differences in broiler chickens fed with 3% wheat bran for two weeks (*p* > 0.05, Table 6).

## 4. Discussion

In China, wheat bran for feed use is graded by the CP contents; Grade 1 wheat bran have ≥17% CP and Grade 2 wheat bran have ≥15% CP. However, our data showed that the CP contents of wheat bran displayed very few variations; in contrast, the content of starch and fibrous components and IVDE had greater CVs. This finding was consistent with data from the Feedpedia [13]. In previous studies, the effective energy of wheat bran has been determined to be the AME or nitrogen-corrected AME (AMEn). Ning et al. [14] analyzed the AME of five wheat brans in the 12–14-day-old AA broilers, and the values of AME ranged from 1637 to 1845 kcal/kg DM. In a study conducted by Gallardo et al. [19], the AMEn of the wheat bran was determined to be 1395 kcal/kg DM in 15–18-day-old Cobb 500 broilers. In slow-growing broilers, the AME of the wheat bran was reported to be 2303 kcal/kg DM in the 15–25-day-old birds and 2479 kcal/kg DM in the 35–45-day-old birds, respectively [20]. Huge variations in the AME have been revealed across different studies. Apart from compositional differences in each wheat bran sample, the breed and the age of the experimental animals, as well as the fecal collection procedures, might also contribute to large variations in the values of the AME. To analyze the compositional differences in wheat bran, the in vitro digestion was used in the current study to determine the AME of different wheat bran. The IVDE has previously been demonstrated to accurately predict the AME of feed in chickens [17,21]. As shown, the IVDE ranged from 1751 to 2721 kcal/kg DM for the wheat bran collected in this study. It could be speculated from the equation IVDE (kcal/kg DM) = 2966 + 23.65 × Starch − 26.60 × NDF that the effective energy of the wheat bran was affected by the contents of starch and NDF. The PCA also revealed that the NDF was one of the key features to cluster wheat brans into different groups. Therefore, fiber contents were used to classify wheat brans into three groups in the current study, with the LF group having an NDF count of less than 40% and the HF group having an NDF count of over 50%.

When fibrous ingredients were added to the diets directly without balancing the other nutrients, the feed intake of broilers was increased to meet their need for energy, because of the dilution effect of the wheat bran [5,22]. An increased feed intake physically stimulated digestion and the development of the gizzard [2,4,22]. Several studies have shown that the inclusion of 3% wheat bran augmented gizzard weight and increased pancreatic amylase and trypsin activities in broilers, consequently improving the digestion of CP, EE, and organic matter, as well as THE growth performance [3,6]. To exclude the interference from feed intake, all the experimental diets were formulated to be isocaloric to maintain the same level of energy concentration in the current study; therefore, similar levels of ADFI and ADG were found in the different treatment groups. Due to their low digestibility, adding wheat brans, especially the HF wheat bran, significantly decreased the digestibility and the retention of GE and CP as well. These drawbacks of the HF wheat bran would have decreased the growth performance, if the trials had lasted for a longer period of time. However, the inclusion of the LF wheat bran did not affect the retention for GE and CP. The LF wheat bran also improved the broilers’ digestion ability for CP and NDF when the birds were switched onto the control diet. These findings suggested that a moderate amount of the LF wheat bran in the young birds’ diet could benefit the CP and NDF digestion ability and possibly growth performance in broilers. This is consistent with previous findings that wheat bran increased the digestion ability for sorghum [11] and barley [6,12]. These findings might also benefit the fast accumulation of body weight in a later growth stage, because an improvement in growth performance was noted in birds fed with wheat brans until the marketed age [3,11,23].

Due to the increased ability of fiber digestion in the birds fed with wheat bran, changes in the cecal microbiome were expected. In this study, 16S rDNA sequencing was used to profile the cecal microbiota. The data revealed that the microbes utilizing complex carbohydrates, such as *Faecalibacterium* and *Lachnospiraceae*, accounted for more than 60% of the cecal microflora, and high abundances of GH and glycosyltransferase were also shown in the KEGG annotation. These findings were highly consistent with the results of whole genome sequencing analyses [24,25]. It can be concluded that the microbes in the cecum of broilers also harbored a strong ability to hydrolyze dietary fibers. According to the α and β-diversity analyses, adding 3% LF wheat bran to the broilers’ diet reduced rare species and resulted in a more even cecal microbiome in broiler chickens. As shown in Figure 5, the LF wheat bran specifically increased the relative abundance of *Barnesiella* at the genus level. Several studies have shown that an increased abundance of *Barnesiella* correlated with reduced intestinal lesions when broiler chickens were fed with *Clostridium butyricum* or glycerol monolaurate, or received the coccidiosis vaccine [26,27]. In human studies, *Barnesiella* has been shown to augment the production of IFN-γ, thereby regulating the TLR4 signal and immune homeostasis [28,29]. Hence, the LF wheat bran could enhance broilers’ immune function, especially when challenged with enteric pathogens. Although *Lachnospiraceae* was capable of degrading complex carbohydrates and *Barnesiella* had genes to produce SCFAs, abundances of majority GH and cecal SCFAs did not significantly differ in the birds fed with different wheat brans. It seemed that a 2-week dietary inclusion of wheat bran was not sufficient to interfere with SCFAs production in broiler chickens.

## 5. Conclusions

According to their proximate components and the IVDE, the wheat bran from different resources were clustered into the LF, MF and HF groups. Among the chemical constituents, the fibrous components were the key parameters for predicting nutritional values of wheat bran in broiler chickens. The current study demonstrated that adding a moderate amount of the LF wheat bran into the diet could promote the growth of *Barnesiella* in the gut, augmenting the digestion of dietary CP and NDF in broilers. The LF wheat bran with an NDF content of less than 40% were recommended for use in the broilers’ diet.

## Figures and Tables

**Table 1:** Composition and nutritional levels of the experimental diets.

Item	D0–6	D7–21						D22–28
		CON	WB1	WB2	WB3	WB4	WB5	
Ingredients, %								
Corn	50.28	50.28	48.14	48.14	48.14	48.14	48.14	57.59
Soybean meal	38.80	38.80	37.15	37.15	37.15	37.15	37.15	31.50
Wheat bran	0.00	0.00	3.00	3.00	3.00	3.00	3.00	0.00
Soybean oil	4.50	4.50	5.10	5.11	5.21	5.28	5.29	5.50
Corn gluten meal	2.00	2.00	2.00	2.00	2.00	2.00	2.00	2.00
*L*-Lysine HCl	0.25	0.25	0.25	0.25	0.25	0.25	0.25	0.14
*DL*-Methionine	0.20	0.20	0.20	0.20	0.20	0.20	0.20	0.13
*L*-Threonine	0.10	0.10	0.10	0.10	0.10	0.10	0.10	0.03
Valine	0.10	0.10	0.10	0.10	0.10	0.10	0.10	0.01
Phytase	0.02	0.02	0.02	0.02	0.02	0.02	0.02	--
Dicalcium phosphate	1.89	1.89	1.89	1.89	1.89	1.89	1.89	1.43
Limestone	1.06	1.06	1.06	1.06	1.06	1.06	1.06	0.92
Premix ^†^	0.50	0.50	0.50	0.50	0.50	0.50	0.50	0.50
NaCl	0.30	0.30	0.30	0.30	0.30	0.30	0.30	0.25
Bentonite	0.00	0.00	0.19	0.18	0.08	0.01	0.00	0.00
Total	100.00	100.00	100.00	100.00	100.00	100.00	100.00	100.00
Nutrient composition ^‡^								
IVDE, kcal/kg	3500	3500	3500	3500	3500	3500	3500	3660
CP, %	26.14	26.14	25.97	26.02	26.04	25.89	26.02	21.79
NDF, %	9.30	9.30	10.00	10.41	10.37	10.99	11.19	9.20
ADF, %	5.18	5.18	5.37	5.59	5.66	5.68	5.71	4.85

Note: ^†^ The premix provided the following per kilogram of diet: vitamin A, 8000 IU; vitamin D_3_, 1000 IU; vitamin E, 20.0 IU; vitamin K3, 0.80 mg; thiamine, 3.0 mg; riboflavin, 8.0 mg; vitamin B6, 5.0 mg; vitamin B12, 20.0 µg; pantothenic acid, 10.0 mg; nicotinic acid, 40.0 mg; folic acid, 0.60 mg; biotin, 0.20 mg; Cu (as copper sulfate), 8.0 mg; Fe (as ferrous sulfate), 100 mg; Mn (as manganese sulfate), 120 mg; Zn (as zinc sulfate), 100 mg; I (as calcium iodate), 0.70 mg; Se (as sodium selenite), 0.30 mg. ^‡^ The nutrient composition was on a dry matter basis, where IVDE of each diet was calculated. Abbreviations: IVDE, in vitro digestible energy; CP, crude protein; NDF, neutral detergent fiber; ADF, acid detergent fiber.

**Table 2:** The chemical compositions of wheat brans (DM basis, %).

Sample	GE, kcal/kg	IVDE, kcal/kg	Starch	CP	EE	NDF	ADF	Hemicellulose	Ash
WB1	4668	2479	21.48	20.71	4.31	34.95	10.05	24.90	4.97
WB2	4524	2430	26.53	17.63	2.91	42.80	11.85	30.95	5.16
WB3	4625	2133	17.37	19.38	3.84	43.38	12.03	31.35	5.76
WB4	4583	1918	13.23	17.87	3.20	52.11	13.98	38.14	6.37
WB5	4549	1876	16.35	17.62	3.17	55.77	15.21	40.56	6.24
WB6	4644	2721	23.58	22.14	4.68	33.27	9.51	23.76	5.55
WB7	4651	2349	19.07	21.02	3.94	42.56	9.65	32.92	5.63
WB8	4551	1751	8.52	18.75	3.11	53.30	14.52	38.78	6.81
Mean	4599	2207	18.27	19.39	3.65	44.77	12.10	32.67	5.81
SD	55	341	5.76	1.73	0.64	8.34	2.27	6.26	0.62
CV, %	1.19	15.46	31.54	8.91	17.62	18.63	18.75	19.15	10.74

Abbreviations: DM, dry matter; GE, gross energy; IVDE, in vitro digestible energy; CP, crude protein; EE, ether extract; NDF, neutral detergent fiber; ADF, acid detergent fiber; CV, coefficient of variation.

**Table 3:** Growth performance of broilers fed with 3% wheat bran from d 7 to 21.

Treatment	Day 7 BW, g	Day 21 BW, g	ADG, g/d	ADFI, g/d	F/G
CON	190.7	1097.3	64.8	77.6	1.198 ^b^
WB1	190.2	1092.4	64.5	78.2	1.213 ^ab^
WB2	190.0	1076.0	63.3	77.7	1.228 ^a^
WB3	190.8	1081.9	63.7	78.3	1.231 ^a^
WB4	189.5	1059.3	62.1	76.2	1.229 ^a^
WB5	190.7	1079.0	63.5	77.8	1.225 ^a^
SEM	0.4	10.2	0.8	0.9	0.006
*p*-value					
ANOVA	0.200	0.167	0.155	0.409	0.007
LF vs. MF	0.658	0.293	0.281	0.859	0.039
LF vs. HF	0.935	0.067	0.066	0.194	0.069
MF vs. HF	0.521	0.315	0.324	0.170	0.736

Note: Means in the same column with different superscripts differ significantly. Abbreviations: CON is the control group fed with corn–soybean basal meal; WB1, WB2, WB3, WB4, and WB5 are the treatment groups receiving the corresponding 3% wheat bran; LF represents the treatment group which received the LF wheat bran (WB1); MF represents the treatment group which received the MF wheat bran (WB2, WB3); and HF represents the treatment group which received the HF wheat bran (WB4, WB5). ADG, average daily gain; ADFI, average daily feed intake; BW, body weight; F/G, feed to gain ratio.

**Table 4:** Coefficients of total tract nutrient metabolizability when broilers were fed with different experiment diets between d 17 and 20.

Treatment	DM	GE	CP	NDF	ADF	Energy Retention, g/d	CP Retention, g/d
CON	0.709 ^a^	0.734 ^a^	0.652	0.266 ^c^	0.071	330.22 ^a^	16.75
WB1	0.697 ^ab^	0.724 ^ab^	0.651	0.284 ^bc^	0.050	331.54 ^a^	16.72
WB2	0.698 ^ab^	0.722 ^ab^	0.649	0.299 ^abc^	0.104	321.98 ^ab^	16.26
WB3	0.690 ^b^	0.714 ^b^	0.638	0.299 ^abc^	0.090	327.67 ^ab^	16.22
WB4	0.694 ^b^	0.719 ^b^	0.645	0.342 ^a^	0.085	315.27 ^b^	15.81
WB5	0.690 ^b^	0.718 ^b^	0.644	0.319 ^ab^	0.094	315.53 ^b^	15.88
SEM	0.003	0.003	0.005	0.010	0.013	4.32	0.25
*p*-value							
ANOVA	0.001	0.005	0.245	<0.001	0.100	0.039	0.051
LF vs. MF	0.331	0.173	0.185	0.240	0.008	0.216	0.128
LF vs. HF	0.135	0.219	0.252	0.001	0.023	0.005	0.008
MF vs. HF	0.504	0.864	0.819	0.004	0.579	0.039	0.126

Note: Means in the same column with different superscripts differ significantly. Abbreviations: CON is the control group fed with corn–soybean basal meal; WB1, WB2, WB3, WB4, and WB5 are the treatment groups receiving the corresponding 3% wheat bran; LF represents the treatment group which received the LF wheat bran (WB1); MF represents the treatment group which received the MF wheat bran (WB2, WB3); HF represents the treatment group which received the HF wheat bran (WB4, WB5); DM, dry matter; GE, gross energy; CP, crude protein; NDF, neutral detergent fiber; ADF, acid detergent fiber.

**Table 5:** Coefficients of total tract nutrient metabolizability when each group of broilers were on the same diet between d 24 and 27.

	GE	DM	CP	NDF	ADF
CON	0.779	0.741	0.623 ^bc^	0.272	0.182
WB1	0.787	0.745	0.647 ^a^	0.322	0.170
WB2	0.782	0.744	0.637 ^ab^	0.301	0.170
WB3	0.779	0.738	0.634 ^abc^	0.281	0.171
WB4	0.780	0.740	0.626 ^bc^	0.282	0.184
WB5	0.777	0.738	0.622 ^c^	0.278	0.193
SEM	0.003	0.002	0.005	0.012	0.011
*p*-value					
ANOVA	0.188	0.144	0.009	0.059	0.608
LF vs. MF	0.070	0.162	0.073	0.042	0.971
LF vs. HF	0.022	0.049	0.001	0.008	0.201
MF vs. HF	0.495	0.415	0.025	0.379	0.115

Note: Means in the same column with different superscripts differ significantly. Abbreviations: CON is the control group fed with corn–soybean basal meal; WB1, WB2, WB3, WB4, and WB5 are the treatment groups receiving the corresponding 3% wheat bran; LF represents the treatment group which received the LF wheat bran (WB1); MF represents the treatment group which received the MF wheat bran (WB2, WB3); HF represents the treatment group which received the HF wheat bran (WB4, WB5); GE, gross energy; DM, dry matter; CP, crude protein; NDF, neutral detergent fiber; ADF, acid detergent fiber.

**Table 6:** Contents of short chain fatty acids in broilers fed with 3% wheat brans.

Treatment	Acetate, mg/g	Propionate, mg/g	Isobutyrate, mg/g	Butyrate, mg/g	Isovalerate, mg/g	Valerate, mg/g	SCFA, mg/g
CON	5.49	0.53	0.24	1.49	0.21	0.18	8.15
WB1	4.15	0.47	0.17	0.95	0.17	0.12	6.04
WB2	3.85	0.36	0.24	0.92	0.14	0.12	5.64
WB3	3.35	0.31	0.14	0.81	0.13	0.11	4.83
WB4	3.90	0.46	0.13	1.05	0.16	0.13	5.82
WB5	4.26	0.52	0.27	0.98	0.25	0.15	6.43
SEM	0.72	0.09	0.06	0.19	0.03	0.02	1.02
*p*-value	0.518	0.502	0.407	0.261	0.176	0.303	0.429

Abbreviations: CON is the control group fed with corn–soybean basal meal; WB1, WB2, WB3, WB4, and WB5 are the treatment groups receiving the corresponding 3% wheat bran. SCFAs, short chain fatty acids.

## Data Availability

The data presented in this study are available in the article.
